# TIF Guidelines for the Management of Transfusion‐Dependent β‐Thalassemia

**DOI:** 10.1002/hem3.70095

**Published:** 2025-03-05

**Authors:** Khaled M. Musallam, Maria Domenica Cappellini, John B. Porter, Dimitrios Farmakis, Androulla Eleftheriou, Michael Angastiniotis, Ali T. Taher

**Affiliations:** ^1^ Center for Research on Rare Blood Disorders (CR‐RBD), Burjeel Medical City Abu Dhabi United Arab Emirates; ^2^ Department of Public Health & Epidemiology Khalifa University Abu Dhabi United Arab Emirates; ^3^ Division of Hematology/Oncology, Department of Pediatrics Weill Cornell Medicine New York New York USA; ^4^ Unit of Medicine and Metabolic Disease Fondazione IRCCS Ca' Granda Ospedale Maggiore Policlinico Milan Italy; ^5^ Department of Haematology University College London Hospitals London UK; ^6^ Department of Cardiology Athens University Hospital Attikon, National and Kapodistrian University of Athens Medical School Athens Greece; ^7^ Thalassaemia International Federation (TIF) Nicosia Cyprus; ^8^ Department of Internal Medicine American University of Beirut Medical Center Beirut Lebanon

The prospect of patients with transfusion‐dependent β‐thalassemia (TDT), once considered a fatal childhood disorder, has completely transformed over the past 50 years.^1^ This is primarily attributed to the adoption of hemovigilance in transfusion therapy, the development of effective iron chelators, the validation of non‐invasive tools for monitoring organ‐specific iron loading, and the introduction of multidisciplinary care. Regrettably, access to these advances and optimal application of best practices remain largely confined to nations with robust economies, where comprehensive health and social care systems provide universal access to treatment.^2^ Consequently, multimorbidity and shortened survival continue to burden patients in countries with limited resources, where most of TDT patients live.^3^ In high‐income settings, improved survival of TDT did not come without its own “side effect,” where aging allowed several previously unrecognized morbidities to manifest, especially in patients who were exposed to the harmful effects of under‐ or sub‐optimal treatment in the past.^4^, ^5^ Thus, the “gold standard” in TDT care is now fundamentally recognized as being a multidisciplinary approach to management, preferably in expert or reference centers, and with active engagement of patients and their families.^6^

Since its inception in 1986, the Thalassaemia International Federation (TIF) has remained committed to supporting patients/families and patient organizations, healthcare professionals, and policymakers to promote optimal care for patients with thalassemia and other hemoglobinopathies across the world. The preparation, publication, translation, and free distribution of management guidelines is a cornerstone of its educational mission. Widely recognized for their significant impact on the care of patients with TDT, and broadly endorsed by the international medical community, these guidelines serve as a critical resource for healthcare providers and a foundation upon which national policies and practices can be built.

The 5th edition of TIF “Guidelines for the Management of Transfusion‐Dependent β‐Thalassemia (TDT)” has now become available (available for free download at: https://thalassaemia.org.cy/tif-publications/).^7^ In keeping with the many and multiple unmet needs of patients in different geographies, the guidelines have been carefully crafted to offer evidence‐based recommendations (key points and recommendations from select chapters on diagnosis and disease management are provided in Table 1, and a summary of monitoring recommendations is provided in Supporting Information S1: Table S1) while also suggesting solutions and pathways for care in resource‐limited settings. Recommendations are provided by a global and diverse group of experts with decades of experience in patient care, through 17 interconnected chapters that echo the need of “true” multidisciplinary care throughout the patient journey from childhood to adulthood. The guidelines also include a chapter dedicated to the value of patient engagement, emphasizing the transformative power of patients as informed and effective advocates for their own needs. Additionally, patients have contributed to the review of various other chapters, ensuring that issues such as lifestyle and mental health are comprehensively addressed from the patients' perspective.

**Table 1:** Key points and recommendations from chapters covering diagnosis, blood transfusion, iron overload and chelation, hematopoietic cell transplantation, gene manipulation, and novel disease‐modifying agents.

**Genetic Basis, Pathophysiology, and Diagnosis**
*Origa R, Sheth S, Sollaino MC, Rivella S*
1. A diagnosis of thalassemia should be considered in all those who have hypochromic microcytic anemia (**Grade C, Class I**). 2. In the diagnostic work‐up for hypochromic microcytosis, iron deficiency anemia should always be excluded (**Grade C, Class I**). 3. Molecular analysis is not required to confirm the diagnosis of a β‐thalassemia carrier, but it is necessary to confirm the α‐thalassemia carrier status (**Grade C, Class I**). 4. An α‐globin gene triplication or quadruplication should be taken into consideration in heterozygous β‐thalassemia subjects with a β‐thalassemia intermedia phenotype (**Grade C, Class I**). 5. Hematological parameters, including red cell indices and morphology, followed by separation and measurement of hemoglobin fractions, are the basis for the identification of β‐thalassemia carriers (**Grade C, Class I**). 6. Since the prevalent pathogenic variants of the β‐globin gene are limited in each at‐risk population, a PCR method designed to detect the common specific mutation simultaneously should be used initially (**Grade C, Class IIa**). 7. β‐globin gene sequence analysis may be considered first if the affected individual is not of an ancestry at high risk or if targeted analysis reveals only one or no pathogenic variant (**Grade C, Class IIa**). 8. Methods that may be used to detect rare or unknown deletions include Southern blotting (now fallen into abeyance), quantitative PCR, long‐range PCR and, above all, MLPA (**Grade C, Class IIa**). 9. Considerations of phenotype should not only be based on genotype but should take clinical presentation and disease severity as observed over a duration of time (**Grade C, Class IIb**). 10. Patients who receive six or more red blood cell units over 6 months with ≤6‐week transfusion‐free period or receiving frequent transfusions for >1 year can be classified as TDT for purposes of management approach or clinical trial eligibility (**Grade C, Class IIb**).

**Blood Transfusion**
*Shah F, Wood E, Maggio A*
1. Confirm the diagnosis of thalassemia, perform appropriate clinical and laboratory assessment, and obtain informed consent before initiation of transfusion (**Grade C, Class IIa**). 2. The decision to initiate a long‐term regular transfusion regimen should not only be driven by patient genotype or hemoglobin level, and should not be driven by a transient drop in hemoglobin due to an intercurrent infection. It should consider the current clinical phenotype of the patient and anticipated short‐ and longer‐term outcomes and should be taken in discussion with the patient or parents (**Grade C, Class IIa**). 3. Use careful donor selection and screening, favoring voluntary, regular, and non‐remunerated blood donors (**Grade C, Class IIa**). 4. Before the first transfusion, perform extended red cell antigen typing of patients at least for Rh C, c, D, E, e and Kell (K, k), and if available a full red cell pheno/genotype (**Grade C, Class IIa**). 5. At each transfusion, give ABO, Rh(D) compatible blood. The goal for all blood transfusions to thalassemia patients is units that are also matched for C, c, E, e, and Kell antigens (**Grade C, Class IIa**). 6. If units are optimally matched, then fresher units can be chosen over older units in less regulated blood banks but in highly regulated blood banks, a first‐in first‐out principle is reasonable (**Grade C, Class IIb**). 7. Before each transfusion, perform a screen for new antibodies and an indirect antiglobulin test crossmatch, or in centers that meet regulatory requirements, perform an electronic crossmatch where allowed (**Grade C, Class I**). 8. Use leukodepleted packed red cells. Pre‐storage filtration is strongly recommended, but blood bank pretransfusion filtration is acceptable. Bedside filtration is only acceptable if there is no capacity for pre‐storage filtration or blood bank pretransfusion filtration (**Grade C, Class I**). 9. Use washed red cells for patients who have severe allergic reactions (**Grade C, Class IIa**). 10. Transfuse every 2–5 weeks, targeting a pretransfusion hemoglobin of 9.5–10.5 g/dL (**Grade C, Class I**). 11. A higher target pretransfusion hemoglobin level of 10–11 g/dL (or as high as practicable) may be appropriate for patients with heart disease including pulmonary hypertension, clinically significant extramedullary hematopoiesis, or other medical conditions, and for those patients who do not achieve adequate suppression of bone marrow activity at the lower hemoglobin level (**Grade C, Class I**). 12. Maintaining pretransfusion hemoglobin at 10 g/dL is important in reducing both maternal cardiovascular stress and improving fetal outcomes in pregnant women (**Grade C, Class I**). 13. Keep the post‐transfusion hemoglobin below 13–15 g/dL (**Grade C, Class IIa**). 14. Hemovigilance and adverse events reporting are key to the safety of blood transfusion. Keep a record of red cell antibodies, transfusion reactions, and annual transfusion requirements for each patient (**Grade C, Class IIa**).

**Iron Overload and Chelation**
*Porter JB, Wood JC, Coates TD*
1. Uncontrolled transfusional iron overload increases the risks of heart failure, endocrine damage, liver cirrhosis and hepatocellular carcinoma in TDT (**Grade C**). 2. The absolute change in total body iron in response to transfusion and chelation can be calculated from the change in LIC. The direction of change in body iron in response to transfusion and chelation can usually but not always be estimated from the trend in SF. Cardiac storage iron concentration is directly related to the risk of heart failure, which can be reliably estimated by mT2*. Cardiac iron accumulates later than liver iron, and is rare before the age of 8 years or 5 years of transfusion on those beginning regular transfusion later in life, affecting a subset of patients; while chelation of storage iron from the liver tends to be faster than from the myocardium (**Grade B, Class I**). 3. Serial SF measurement is indicated in all TDT patients, to be conducted regularly at least every 3 months or at shorter/longer frequencies as needed based on iron overload level and iron chelation modification needs (**Grade B, Class I**). 4. Hepatic and cardiac MRI for the assessment of LIC and mT2* should be performed annually starting at the age of 8–10 years (or earlier if feasible without sedation need). Shorter/longer frequencies can be adopted as needed based on iron overload level and iron chelation modification needs. Reading and interpretation should be done by trained staff or outsourced third‐party vendors, using a validated method with appropriate calibration and MRI acquisition techniques (**Grade B, Class IIa**). 5. LIC determination should be considered in patients whose SF levels are high (>4000 ng/mL) or deviate from expected trends, or when new chelating regimes are being used. LIC assessment cannot predict (or replace) mT2* assessment (**Grade C, Class IIb**). 6. TDT patients should undergo regular assessment for growth, development, and organ function (including the heart, liver, and endocrine glands) as per recommendations in the respective chapters in these guidelines. 7. Chelation therapy is an effective treatment modality in improving survival, decreasing the risk of heart failure, and decreasing morbidities from transfusion‐induced iron overload (**Grade C, Class I**). 8. Chelation therapy at the correct doses and frequency can balance iron excretion with iron accumulation from transfusions (**Grade A, Class I**). 9. Prevention of iron accumulation using chelation therapy is preferable to rescue treatment because iron‐mediated damage is often irreversible, and removal of storage iron by chelation is slow—particularly after it has escaped the liver (**Grade B, Class I**). 10. Response to chelation is dependent on the dose applied and the duration of exposure (**Grade A, Class I**). 11. Response to chelation is affected by the rate of blood transfusion (**Grade B, Class I**). 12. Chelation therapy removes myocardial storage iron slowly (months or years) (**Grade A, Class I**). 13. Chelation can reverse iron‐mediated cardiac dysfunction rapidly (within weeks) by rapid chelation of labile iron, if 24‐hour chelation cover is achieved (**Grade B, Class IIa**). 14. The optimal chelation regimen and dosing depend on approved local indications and prescribing information of individual chelators, must be tailored for the individual, and will vary based on the current clinical situation and iron overload profile (**Grade A, Class I**). 15. Overchelation increases side effects from chelation therapy, and doses should therefore be decreased as serum ferritin or liver iron levels fall (demonstrated most clearly with deferoxamine) (**Grade B, Class I**). 16. Patients receiving iron chelation should be closely monitored for unwanted adverse effects and their management including dose modifications/interruptions according to local prescribing information (**Grade A, Class I**). 17. Chelation therapy will not be effective if it is not taken regularly—a key aspect of chelation management is to work with patients and their families to optimize adherence (**Grade B, Class I**).

**Hematopoietic Cell Transplantation**
*Pinto VM, Gambella M, Sodani P, Oevermann L, Angelucci E*
1. HCT should be offered to TDT patients and their parents at an early age, before complications due to iron overload develop, if an HLA identical donor is available (**Grade B, Class I**). 2. Either bone marrow or cord blood from an HLA‐identical sibling can be used (**Grade B, Class I**). 3. A matched unrelated donor can be used, provided that high compatibility criteria for both HLA class I and II loci are met (**Grade B, Class I**). 4. Haploidentical HCT shows promising results but should be considered only in experienced HCT centers in the context of well‐designed clinical trials (**Grade B, Class IIb**). 5. Myeloablative conditioning regimens should always be used for standard transplantation (**Grade B, Class I**). 6. Special attention is required for adult patients (**Grade B, Class I**). 7. Post‐transplant care should include all transplant and thalassemia‐related complications (**Grade B, Class I**). 8. Only thalassemia‐expert transplant centers should perform HCT, always in strict connection with thalassemia reference centers (**Grade C, Class I**). 9. In TDT patients, HCT is cost‐effective when compared to life‐long supportive therapy (**Grade B, Class I**).

**Gene Manipulation**
*Locatelli F, Algeri M*
1. The outcomes achieved with gene therapy approaches in experimental studies, along with the initial regulatory approval of these treatments, are beginning to reshape the landscape of potentially curative options for TDT, redefining particularly the role of allogeneic transplantation. 2. In this regard, it is critical to emphasize that pediatric patients up to the age of 14 years undergoing allogeneic HCT from HLA‐identical sibling donors demonstrate excellent clinical outcomes. Within this framework, allogeneic HCT remains the preferred curative intervention and warrants thorough consideration, particularly in regions outside the USA where no gene therapy is yet commercially accessible for patients below 12 years (**Grade B, Class I**). 3. Recent registry data further suggest that, in highly specialized transplantation centers, outcomes of allogeneic HCT using fully HLA‐matched (10/10) unrelated volunteers are comparable to those achieved with HLA‐identical sibling donors. Nonetheless, the decision to pursue HCT from unrelated registry donors in pediatric patients with TDT should be meticulously discussed with the families (**Grade B, Class IIa**). This discussion should encompass the potentially higher risk of immune‐mediated complications associated with unrelated donors, along with the anticipated broader availability of gene addition and gene editing strategies in the near future. 4. For patients aged 14 years and older or for those who do not have an HLA‐identical family donor, gene therapy instead constitutes an optimal therapeutic option (**Grade C, Class IIa**): Findings from the beti‐cel exa‐cel registrational studies did not reveal any specific clinical characteristics associated with an improved safety and efficacy profile. Results were comparable between adolescent and adult subjects, with no observed differences in outcomes based on iron overload status, genotype, transfusion burden, or other factors. Consequently, there are no absolute indicators, within the studied population, to determine which patients should be prioritized for treatment with either one of the two products. In addition, the unique challenges posed by autologous gene therapy approaches require new frameworks that cannot be directly extrapolated from the experiences and knowledge gained through allogeneic transplantation. 5. Beti‐cel is currently available only in the USA, where it is FDA‐approved for pediatric and adult subjects with TDT, irrespective of the patient's age and genotype. Exa‐cel has been recently approved by regulatory agencies in Europe (EMA), North America (FDA), the UK, Bahrain, and Saudi Arabia for TDT patients aged 12 years and above, without an upper age limit. Despite the absence of age limitations, during the decision‐making process, it is advisable to conduct an initial selection based on the primary inclusion/exclusion criteria used in the Northstar and CLIMB THAL‐111 studies, which supported the submission for regulatory approvals. At least in the initial phases, it is recommended not to consider the treatment of patients who do not meet the eligibility characteristics defined by the registrational studies (**Grade C, Class IIb**): With respect to patient's age, the safety and efficacy of beti‐cel was studied in clinical trials that enrolled patients between the ages of 4 and 35 years old, while the exa‐cel study was conducted on patients aged 12–35 years. Within this age range, the ideal candidate is a patient who exhibits the following characteristics (**Grade C, Class IIa**): − Significant transfusion history (at least 100 mL/kg or at least 10 units/year of packed red blood cells). − Adequate control of iron overload (LIC ≤ 7 mg/g dry liver weight and mT2* > 20 ms). − Normal ventricular function and respiratory function tests. − Absence of significant hepatosplenomegaly. − Absence of gallstone disease. − Potential for effective fertility preservation. − High level of personal motivation. − This type of patient profile is associated with the highest likelihood of success and the lowest risk of treatment‐related toxicity. Moreover, this category of patients is most likely to benefit from such an intervention due to the following factors: ∘ A greater number of transfusion‐free years if treatment is successful, contributing to a longer life expectancy. ∘ Lower risk of exacerbation or chronicity of prior damage related to the underlying disease. ∘ High probability of successful mobilization and collection of hematopoietic stem cells (reducing the need of additional cycles beyond the initial one). ∘ Decreased risk of delayed engraftment and related complications. ∘ Minimization of treatment‐related infertility risk, positively impacting the post‐treatment quality of life. Patients presenting with at least one of the characteristics listed below should also be considered for treatment, ideally within a short timeframe (24 months), provided that the treatment is administered in centers with proven expertise in managing TDT patients using allogeneic HCT or gene therapy approaches (**Grade C, Class IIb**): – Age between 35 and 45 years. – Moderate‐to‐severe iron overload (LIC > 7 and <15 mg/g dry liver weight and mT2* < 20 and >15 ms). – Rare erythrocyte phenotypes or a history of alloimmunization, which could lead to foreseeable difficulties in the identification and long‐term availability of suitable red blood cell units (provided that transfusion support can be ensured during treatment phases). – Proven intolerance to iron‐chelating drugs (due to allergic reactions or excessive side effects) that would likely result in a rapidly progressive worsening of iron overload.

**Novel Disease‐Modifying Agents**
*Musallam KM, Sheth S, Cappellini MD, Kuo KHM, Rivella S, Maggio A, Viprakasit V, Taher AT*
1. Luspatercept is recommended in TDT adults (≥18 years) to achieve transfusion burden reduction (**Grade B**, **Class I**). 2. The following patient subgroups may be prioritized for luspatercept treatment (**Grade C, Class IIb**): Patients receiving moderate transfusion regimens (≤4 packed red blood cell units/month). Patients with non‐β°/β^0^ genotype. Splenectomized patients. Patients unable to sustain transfusion regimen for target hemoglobin level Patients with progressive iron overload (nonadherence, poor tolerance/response to iron chelation therapy). 3. Luspatercept treatment dosing should follow local prescribing information; otherwise, the below guidance should be followed (**Grade B**, **Class I**): Starting dose of 1 mg/kg subcutaneously every 3 weeks. Dose increase to 1.25 mg/kg if patient has no reduction in transfusion burden after at least 2 consecutive doses (6 weeks) of 1 mg/kg. − Per US FDA product label. In the EMA label, <33% transfusion burden reduction is required. Treatment discontinuation if patient has no reduction in transfusion burden after 3 consecutive doses (9 weeks) of 1.25 mg/kg (minimum overall treatment duration of at least 15 weeks). Treatment interruption if pre‐dose hemoglobin is ≥11.5 g/dL in the absence of transfusions. Treatment can be restarted when hemoglobin is ≤11 g/dL. Dose decrease if increase in hemoglobin is >2 g/dL within 3 weeks and in the absence of transfusions (decrease 1.25 mg to 1.0 mg, 1 mg to 0.8 mg, 0.8 mg to 0.6 mg, interrupt if 0.6 mg). − Luspatercept 0.6 mg/kg dose is approved for TDT patients by the US FDA label only; the EMA label states that the lowest dose should be 0.8 mg/kg. 4. Luspatercept treatment adverse event monitoring and management should follow local prescribing information; otherwise, the below guidance should be followed (**Grade B**, **Class I**): Mild adverse events are generally manageable with over‐the‐counter analgesics/medications. For patients who experience persistent high‐grade adverse events: − Treatment may be interrupted until the adverse event resolves. − Dosage may be modified. − Treatment may be discontinued. − Individual patient assessment and close monitoring are essential. Treatment should be discontinued for grade 3 or 4 hypersensitivity reactions. Thromboembolic events: considering the higher number of thromboembolic events observed in luspatercept‐treated versus placebo patients in the BELIEVE trial, patients should be monitored for signs and symptoms of thromboembolic events and treatment instituted promptly. Thromboembolic risk assessment and prophylaxis in high‐risk patients are advised in patients with β‐thalassemia (especially splenectomized adults) irrespective of luspatercept therapy. Hypertension: patients treated with luspatercept had an average increase in systolic and diastolic blood pressure of 5 mmHg from baseline: − Treatment must be started only if the blood pressure is adequately controlled. − Blood pressure should be monitored before each luspatercept administration. − Luspatercept dose may require adjustment or may be delayed, and patients should be treated for hypertension. EMH: cases of EMH were documented during luspatercept therapy across β‐thalassemia trials. Patients should be monitored at initiation and during treatment with luspatercept for signs and symptoms of EMH masses (paraspinal localization being the most concerning), especially in non‐transfusion‐dependent or sub‐optimally treated transfusion‐dependent patients who are naturally at higher risk of EMH. 5. Application of transfusion reduction during luspatercept treatment (i.e., reduction of the number of transfused units versus increasing the transfusion visit interval) and any alternate definitions of transfusion reduction response (e.g., ≥20%) should follow the physician's discretion (**Grade C, Class IIb**). 6. Patients receiving luspatercept should continue to be monitored and managed for iron overload, with necessary adjustments based on changes in the rate of iron intake (**Grade C, Class IIb**). 7. The off‐label clinical use and repurposing of drugs with a disease‐modifying effect based on available literature in TDT should follow institutional policies and ethical standards (**Grade C, Class IIb**).

*Note*: Chapter authors are also indicated. Please refer to the original guidelines publication for further information including chapters dedicated to management of specific complications (cardiovascular disease, liver disease, growth abnormalities, endocrine disease, bone disease, and other complications), fertility and pregnancy, oral and dental care, nutrition, lifestyle, quality of life, psychological support, multidisciplinary care and reference centers, and patient engagement. Adapted with permission from Taher et al. [7].

**Abbreviations:** beti‐cel, betibeglogene autotemcel; EMA, European Medicines Agency; EMH, extramedullary hematopoiesis; exa‐cel, exagamaglogene autotemcel; FDA, Food and Drug Administration; HCT, hematopoietic cell transplantation; LIC, liver iron concentration; MLPA, multiplex ligation‐dependent probe amplification; MRI, magnetic resonance imagining; mT2*, myocardial T2*; PCR, polymerase chain reaction; SF, serum ferritin; TDT, transfusion‐dependent β‐thalassemia.

**Level of evidence:** Grade A, data derived from multiple randomized clinical trials or meta‐analyses; Grade B, data derived from a single randomized clinical trial or large non‐randomized studies; Grade C, consensus of the experts and/or small studies, retrospective studies, or registries. **Class of recommendation**: Class I, there is evidence and/or general agreement that a given treatment or procedure is beneficial, useful, and effective; Class II, there is conflicting evidence and/or a divergence of opinion about the usefulness/efficacy of the given treatment or procedure; Class IIa, the weight of evidence is in favor of usefulness/efficacy and therefore should be considered; Class IIb, The usefulness/efficacy is less well established by evidence/opinion and, therefore, may be considered; Class III, there is evidence or general agreement that the given treatment or procedure is not useful/effective, and in some cases may be harmful.

The advent of groundbreaking therapies for TDT in the past decade including curative gene therapy (insertion and editing) approaches as well as disease‐modifying treatments approved for clinical use, have been met with great excitement and promise for a brighter future for patients worldwide. Dedicated chapters in the new guidelines present fundamentals of the design, development, and potential place in therapy for these novel agents, while also calling for further real‐world evidence to address knowledge gaps. As previously stressed, the vast majority of patients are still unable to access such developments. This disparity highlights the urgent need to devise pragmatic and actionable solutions for countries, particularly those with high disease prevalence, to prioritize disease‐specific policies for prevention and optimized care.

We strongly encourage healthcare professionals who follow these guidelines to advocate for their full adoption and implementation within their institutions and alert their national competent health authorities about their immense value. Evidence‐based practices are vital for enabling early diagnosis and effective management, a basic human right of all patients, while at the same time contributing to safeguarding the sustainability of healthcare systems, which are greatly threatened consequent to immense geopolitical, economic, environmental, and public health crises.

## AUTHOR CONTRIBUTIONS

All authors contributed to conceptualization and manuscript drafting or critical review.

## CONFLICT OF INTEREST STATEMENT

K. M. M. reports consultancy fees from Novartis, Bristol Myers Squibb (Celgene Corp), Agios Pharmaceuticals, CRISPR Therapeutics, Vifor Pharma, Novo Nordisk, and Pharmacosmos; and research funding from Agios Pharmaceuticals and Pharmacosmos. M. D. C. reports consultancy fees from Novartis, Bristol Myers Squibb (Celgene Corp), Vifor Pharma, and Vertex Pharmaceuticals; and research funding from Novartis, Bristol Myers Squibb (Celgene Corp), La Jolla Pharmaceutical Company, Roche, Protagonist Therapeutics, and CRISPR Therapeutics. J. B. P. reports honoraria from Agios Pharmaceuticals, bluebird bio, Celgene (Bristol Myers Squibb), La Jolla Pharmaceuticals, Protagonism, Silence Therapeutics, and Vifor; and is a consultant for Agios Pharmaceuticals, bluebird bio, and Celgene (Bristol Myers Squibb). D. F. reports speaker honoraria, consultation fees, and/or grants from Abbott, AstraZeneca, Bayer, Boehringer Ingelheim, Leo, Myocardial Solutions, and Roche. A. T. T. reports consultancy fees from Novo Nordisk, Bristol Myers Squibb (Celgene Corp), Agios Pharmaceuticals, Pharmacosmos, and Roche; and research funding from Novo Nordisk, Bristol Myers Squibb (Celgene Corp), Agios Pharmaceuticals, Pharmacosmos, and Roche. All competing interests are outside the present work. The remaining authors have no conflicts of interest to disclose.

## CONSENT TO PARTICIPATE

Not applicable.

### ETHICS STATEMENT

Not applicable.

### FUNDING

None.

## Supporting information

Supporting information.

## Data Availability

Data sharing is not applicable to this article as no new data were created or analyzed.
