# Supplementary material for: TIF Guidelines for the Management of Transfusion‐Dependent β‐Thalassemia
Source: Hemasphere. 2025 Mar 5;9(3):e70095. doi: 10.1002/hem3.70095 (PMC11880825; doi:10.1002/hem3.70095)
Supplement: Supplementary file 1 — Supporting information. [file HEM3-9-e70095-s001.docx]

**SUPPORTING INFORMATION**

**Table S1. Summary of monitoring recommendations.** The below table summarizes routine monitoring recommendations for morbidities and manifestations of transfusion-dependent β-thalassemia featured in the 5^th^ edition of the Thalassaemia International Federation (TIF) ‘Guidelines for the Management of Transfusion-Dependent β-Thalassemia (TDT)’. These represent screening (first level) assessments. Additional assessments may be required to confirm the diagnosis of specific complications. The frequency of assessment may also need to be adjusted (lower/higher) based on previous findings. Further details on these recommendations and their grading can be found in respective chapters in the original guidelines publication, along with recommendations for special scenarios and populations such as fertility and pregnancy. Adapted with permission from Taher AT, Farmakis D, Porter JB, Cappellini MD, Musallam KM. *Guidelines for the Management of Transfusion-Dependent β-Thalassaemia.* 5th ed. Nicosia, Cyprus: Thalassaemia International Federation; 2025.

| **WHAT** | **WHEN/WHO*** |
| --- | --- |
| **Diagnosis** | |
| Clinical examination and family history, complete blood count with red cell indices and morphology, iron studies, and capillary electrophoresis or HPLC | At suspicion of thalassemia in patients with hypochromic microcytic anemia |
| Molecular studies to confirm β-genotype and α-thalassemia status, HLA typing | At diagnosis |
| **Blood transfusion** | |
| Extended red cell antigen typing at least for Rh C, c, D, E, e, and Kell (K, k), and if available, a full red cell pheno/genotype | Before first transfusion |
| New antibodies and indirect antiglobulin test crossmatch or electronic crossmatch | Before each transfusion |
| Hemovigilance and adverse events monitoring and reporting | Ongoing with transfusion |
| Hemoglobin | Before/after each transfusion |
| Blood units, volume, and hematocrit | With each transfusion |
| **Iron overload and chelation** | |
| Calculated transfusional iron intake | Annual |
| Serum ferritin | At least every 3 months, with the start of transfusion therapy |
| LIC (MRI)† | Annually, starting 8-10 years |
| Myocardial T2* MRI† | Annually, starting 8-10 years |
| Iron chelation adverse event monitoring as provided with the drug when receiving authorization from internationally or nationally recognized competent regulatory authorities (e.g., US FDA, EMA) | Ongoing with iron chelation therapy |
| Iron chelation adherence monitoring | Ongoing with iron chelation therapy |
| **Cardiac disease** | |
| Echocardiography (including TRV) | Annually, starting 10 years |
| Electrocardiogram | Regular as clinically indicated |
| VTE risk assessment | In medical and surgical settings based on local guidelines, especially in older patients who are splenectomized, with low pretransfusion hemoglobin, or pregnant |
| **Liver disease** | |
| AST, ALT, alkaline phosphatase, γGT, and bilirubin | Every 3 months |
| Prothrombin time and albumin | Every 3 months, in patients with severe hepatic impairment |
| Hepatic ultrasound | Annually, starting 18 years  Every 6 months, in patients with advanced liver damage, cirrhotic patients, and in patients with severe hepatic iron overload, age >45 years, or chronic viral hepatitis |
| Alpha fetoprotein | Every 6 months, in cirrhotic patients and in patients with severe hepatic iron overload, age >45 years, or chronic viral hepatitis |
| Transient elastography and fibrosis-4 score (in non-splenectomized patients) to assess for fibrosis/cirrhosis | In patients with advanced liver damage |
| **Height and growth disorders** | |
| Clinical and auxological evaluation including weight, height, BMI, height when sitting, growth rate/velocity (charted), and Tanner stage | Every 6 months, starting from patient first access at the treatment center, until adult height achievement and completion of pubertal development |
| **Disorders of pubertal development** | |
| Testicular volume, growth rate, and Tanner stage | Every 6 months, starting from the normal age of puberty, until the completion of pubertal growth |
| **Female hypogonadism** | |
| Menses rhythm for oligo/amenorrhea | Every 6 months |
| FSH, LH, estradiol, and pelvic ultrasound | In patients with oligo/amenorrhea |
| **Male hypogonadism** | |
| Testosterone, FSH, and LH | Annually, starting puberty |
| **Hypothyroidism** |  |
| FT4 and TSH | Annually, starting 9 years  Every 6 months, in patients with suboptimal iron overload management  Every 3 months, in patients receiving drugs interfering with thyroid function (e.g., amiodarone) |
| **Glucose metabolism disorders** | |
| Fasting blood glucose levels and/or blood glucose during OGTT, and HOMA-IR index | At least every 2 years, from 10-18 years  Annually, starting 18 years |
| Serum fructosamine | Periodic (e.g., every 6-12 months) |
| **Hypocorticosurrenalism** | |
| Sodium, potassium, and ACTH/cortisol at 8 a.m. | Annually, starting adolescence |
| **Hypoparathyroidism** | |
| Serum calcium (corrected for albumin) and phosphorus | Annually, starting 10 years |
| PTH, magnesium, serum creatinine, 25OH vitamin D, and 24-hour urine calcium | In patients with hypocalcemia |
| **Growth hormone deficiency (adults)** | |
| IGF-1 level | Annually, starting 25 years or earlier in case of severe iron overload and other pituitary deficits |
| **Bone disease** | |
| BMD by DXA | Every 1-2 years, starting 10 years |
| **Cholelithiasis** | |
| Gall blader ultrasound | As part of routine hepatic ultrasound |
| **Malignancy (other than liver)** | |
| Screening for solid and hematologic malignancies | Per local screening standards and guidelines‡ |
| **Skin manifestations and leg ulcers** | |
| Skin inspection | Every visit |
| **Renal disease** | |
| Serum creatinine and urine analysis with spot ratio of protein/creatinine | Every 6 months |
| Serum calcium, phosphorus, magnesium, uric acid, and 24-hour urine collection for protein/creatinine and calcium/creatinine ratio | Annually, starting 10 years |
| Renal ultrasound | Every 2 years, starting adolescence and in case of laboratory abnormalities |
| **Splenomegaly** | |
| Spleen size assessment on physical exam | Every visit |
| **Infectious disease** | |
| HBsAg | Annually, in unvaccinated patients |
| Anti-HBs | Annually, in vaccinated patients |
| Anti-HCV | Annually, followed by HCV RNA if positive |
| Anti-HIV | Annually |
| **Oral and dental care** | |
| Dental assessment | Regular |
| **Nutrition** | |
| 25OH vitamin D | Every 6 months, to maintain circulating levels above 30 ng/mL (75 mmol/L) |
| Copper, magnesium, selenium, zinc, folate, vitamin C, vitamin E, cholesterol, and triglycerides | Annually, starting 10 years |
| **Patient reported outcomes** | |
| QOL and psychological well-being | Regular |
| *Recommendations apply to all patients unless otherwise indicated.  †Using validated method with appropriate calibration, acquisition, and processing.  ‡If MRI is done regularly for LIC, images can also be checked for abnormalities and tumors, but this does not replace standard cancer screening guidelines.  Abbreviations: LIC, liver iron concentration; MRI, magnetic resonance imaging; FDA, Food and Drug Administration; EMA, European Medicines Agency; HPLC, high-performance liquid chromatography; TRV, tricuspid valve regurgitant jet velocity; VTE, venous thromboembolism; AST, aspartate transaminase; ALT, alanine transaminase; γGT, gamma glutamyl transferase; FIB-4, fibrosis-4 score; BMI, body mass index; FSH, follicle stimulating hormone; LH, luteinising hormone; FT4 free thyroxine; TSH, thyroid-stimulating hormone; OGTT, oral glucose tolerance test; HOMA-IR, Homeostatic Model Assessment for Insulin Resistance; PTH, parathyroid hormone; ACTH, adrenocorticotropic hormone; 25OH, 25-hydroxy; PTH, parathyroid hormone; IGF-1, insulin-like growth factor 1; BMD, bone mineral density, DXA, dual-energy X-ray absorptiometry; HBsAg, hepatitis B surface antigen; Anti-HBs, hepatitis B surface antibody; HCV, hepatitis C virus; HIV, human immunodeficiency virus; QOL, quality of life. | |
